# Giant cell myocarditis mimicking cardiac amyloidosis with apical sparing pattern in echocardiography: a case report

**DOI:** 10.1093/ehjcr/ytag759

**Published:** 2026-11-13

**Authors:** Shun Sawada, Masanobu Miura, Hideaki Endo, Sadahide Ono, Akihiro Nakamura

**Affiliations:** Department of Cardiology, Iwate Prefectural Central Hospital, 1-4-1, Ueda, Morioka 020-0066, Japan; Department of Cardiology, Iwate Prefectural Central Hospital, 1-4-1, Ueda, Morioka 020-0066, Japan; Department of Cardiology, Iwate Prefectural Central Hospital, 1-4-1, Ueda, Morioka 020-0066, Japan; Department of Pathology, Iwate Prefectural Central Hospital, 1-4-1, Ueda, Morioka 020-0066, Japan; Department of Cardiology, Iwate Prefectural Central Hospital, 1-4-1, Ueda, Morioka 020-0066, Japan

**Keywords:** Giant cell myocarditis, Apical sparing pattern, Mechanical circulatory support, Immunosuppressive therapy, Case report

## Abstract

**Background:**

Giant cell myocarditis typically has a poor prognosis among the various types of myocarditis. Early diagnosis and treatment are essential to save lives. Immunosuppressive therapy is used for giant cell myocarditis; however, evidence in patients on mechanical circulatory support is insufficient.

**Case summary:**

A woman in her 60 s with no history of heart disease or medication was admitted to our hospital because of congestive heart failure. Echocardiography revealed an apical sparing pattern suggestive of cardiac amyloidosis. However, owing to the rapid deterioration of symptoms, an urgent right heart catheterization and myocardial biopsy were performed. Right heart catheterization revealed a cardiac index of 1.20 L/min/m^2^, and rapid myocardial histology indicated inflammatory cell infiltration. She was later diagnosed with giant cell myocarditis with cellular infiltration of irregular multinucleated giant cells in the myocardium. Her condition improved after starting mechanical circulatory support and sequential immunosuppressive therapy, and after successful rehabilitation, she was discharged as an outpatient on Day 183.

**Discussion:**

Early diagnosis and prompt initiation of immunosuppressive therapy are essential in giant cell myocarditis; however, specific findings are lacking. Early myocardial biopsy and prompt mechanical circulatory support initiation should be performed in patients with rapidly deteriorating heart failure presenting with amyloidosis-like echocardiographic findings and in cases where only troponin is elevated and creatine phosphokinase MB is not, considering the possibility of severe oedema. Combination immunosuppressive therapy under mechanical circulatory support is associated with a high infection risk, and starting with single-drug immunosuppressive therapy with careful haemodynamic monitoring is acceptable.

Learning points

- Amyloidosis-like echocardiographic findings in patients with rapidly deteriorating heart failure may help diagnose giant cell myocarditis.
- If troponin is elevated but not creatine phosphokinase MB, 　cytoplasmic troponin may help diagnose myocardial oedema and predict myocardial reversibility.
- Combined immunosuppression under mechanical circulatory support raises infection risk; stepwise escalating from monotherapy may be acceptable.

## Introduction

Myocarditis is a phenotype of inflammatory myopericardial syndrome.^1^ Giant cell myocarditis (GCM) is an extremely rare form that progresses rapidly, with a 3-year rate of death or heart transplantation reaching 85%.^1^

GCM often lacks specific clinical findings. A high index of suspicion, prompt myocardial biopsy^2^ and early initiation of mechanical circulatory support (MCS) and immunosuppressive therapy^3^ are essential to improve prognosis. Although combination regimens such as cyclosporine and steroids improve long-term survival compared with steroid monotherapy,^4^ their intensification must be balanced against the risk of infection under MCS. Herein, we report a case of GCM in which amyloidosis was initially suspected; however, a sudden haemodynamic collapse led to the suspicion of fulminant myocarditis, which was successfully treated with MCS and immunosuppressive therapy.

## Summary figure

**Figure:**
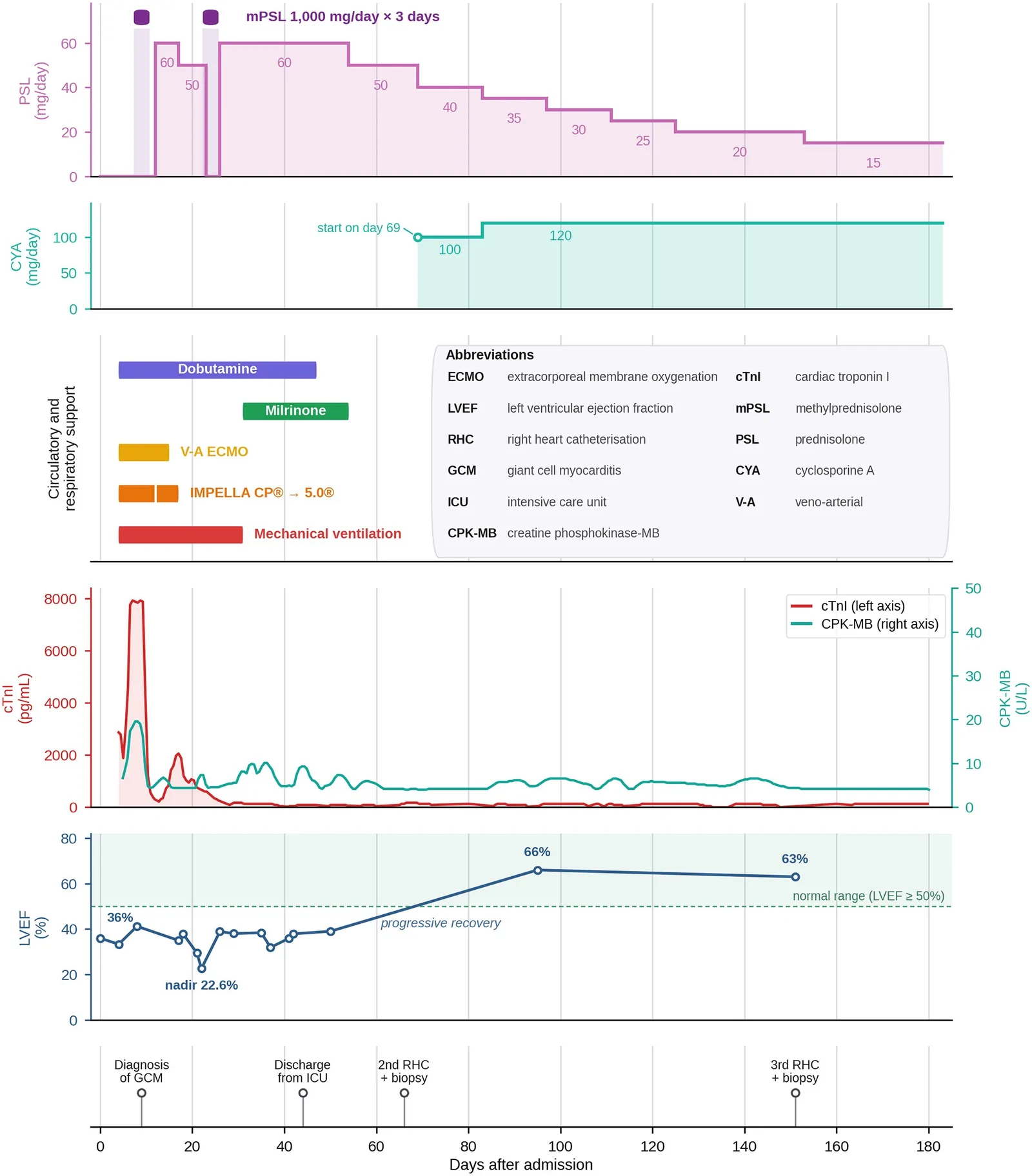
Clinical course of the present patient with fulminant giant cell myocarditis, from admission (Day 0) to hospital discharge (Day 183). All panels share the same horizontal time axis, showing days after admission. From top to bottom, the panels display: (i) the daily oral prednisolone dose (pink step line) together with the two courses of intravenous methylprednisolone pulse therapy (1000 mg/day for 3 days; purple bars) initiated on Days 8 and 23; (ii) the daily dose of cyclosporine A, which was introduced on Day 69 and up-titrated to 120 mg/day by Day 83; (iii) the periods of pharmacological and mechanical support—intravenous dobutamine and milrinone, veno-arterial extracorporeal membrane oxygenation, IMPELLA CP® subsequently upgraded to IMPELLA 5.0® (ECPELLA configuration; white line marks the upgrade), and invasive mechanical ventilation, all initiated on Day 4 when cardiogenic shock developed; (iv) the serial course of cardiac troponin I (red line, left axis) and creatine phosphokinase-MB (green line, right axis), showing a marked early rise in cardiac troponin I with only a modest and transient increase in creatine phosphokinase-MB; (v) the serial left ventricular ejection fraction, which was 36% on admission, declined to a nadir of 22.6% on Day 22 during maximal haemodynamic support, and then subsequently recovered to 39% on Day 50, 66% on Day 95% and 63% on Day 151 (shaded band, normal range ≥50%); and (vi) the major clinical events (open circles), namely the histological diagnosis of giant cell myocarditis on Day 9, discharge from the intensive care unit on Day 44, and the second and third right heart catheterizations with endomyocardial biopsy on Days 66 and 151. Under combined immunosuppressive therapy, the patient was weaned from extracorporeal membrane oxygenation on Day 15 and from IMPELLA 5.0® on Day 17, was extubated on Day 31, and was discharged from hospital on Day 183. The second endomyocardial biopsy on Day 66 showed disappearance of giant cells together with improvement in haemodynamic parameters. **Abbreviations:** CPK-MB, creatine phosphokinase-MB; cTnI, cardiac troponin I; CYA, cyclosporine A; ECMO, extracorporeal membrane oxygenation; ECPELLA, combined veno-arterial ECMO and IMPELLA support; GCM, giant cell myocarditis; ICU, intensive care unit; LVEF, left ventricular ejection fraction; mPSL, methylprednisolone; PSL, prednisolone; RHC, right heart catheterization; V-A, veno-arterial.

## Case presentation

A woman in her 60 s had a fever of 37.9°C a few days before admission and went to her family doctor. She was followed up for cold-like symptoms. The night before admission, she developed chest pain and progressive dyspnoea on exertion, and was referred to our hospital for suspected pneumonia. She had no cardiac history and no regular medication.

Vital signs were normal, and auscultation revealed heart sound III and coarse crackles in the right lower lung region, without signs of low cardiac output. Blood tests indicated significant neutrophilic leucocytosis, high C-reactive protein (CRP) and brain natriuretic peptide levels. She had elevated high-sensitivity cardiac troponin I (hs-cTnI) levels (reference range: 0∼45.2 pg/ml), but not creatine phosphokinase (CPK) and creatine phosphokinase MB (CPK-MB) levels. Although she had no diabetes or liver disease, she also had elevated levels of liver enzymes and high haemoglobin A1c (HbA1c) (*Table 1*). Chest X-ray imaging showed bilateral congestion (*Figure 1A*), and electrocardiography (ECG) revealed low voltage in all leads (*Figure 1B*). Transthoracic echocardiography (TTE) showed diffuse hypokinesis with a left ventricular ejection fraction (LVEF) of 36%, left ventricular hypertrophy (LVH), an apical sparing pattern (*Figure 1C–1E*). TTE also showed an enlarged inferior vena cava of 15 mm without respiratory variability and grade II–III mitral regurgitation. She was admitted with congestive heart failure for further evaluation and treatment.

**Figure 1.**
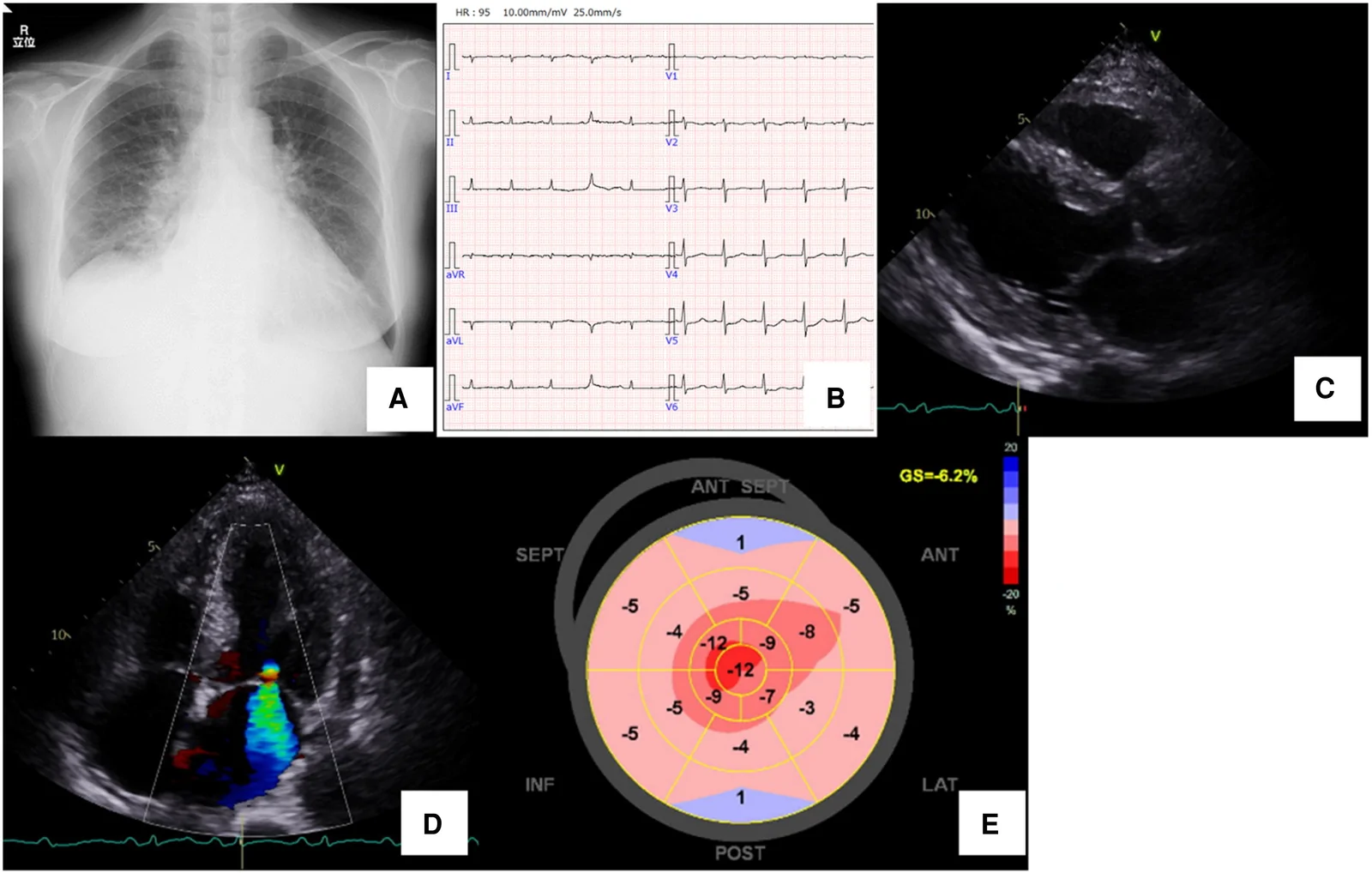
(*A*). The cardiothoracic ratio was 60%. Bilateral hilar shadows were increased, suggesting pulmonary congestion. (*B*). ECG showed a low voltage in whole leads. (*C*). Diffuse left ventricular hypertrophy (LVH). (*D*). Moderate-to-severe mitral regurgitation. (*E*). Apical sparing pattern (apical GLS/basal GLS ratio = 3.0, DcT 83 ms). GLS, global longitudinal strain; DcT, deceleration time.

**Table 1:** Blood test results on admission

WBC	10.02	10^3^/µl
NEUT	81.5	%
LYM	11.9	%
MONO	6.1	%
EO	0.2	%
BASO	0.3	%
RBC	396	10^4^/µg
Hb	12.8	g/dl
PLT	33.1	10^4^/µl
PT-INR	1.09	
APTT	30.8	Sec
CPK	64	U/L
T-Bil	0.7	mg/dl
AST	224	U/L
ALT	184	U/L
LDH	540	U/L
γ-GTP	78	U/L
ALP	231	U/L
AMY	29	U/L
TP	6.1	g/dl
ALB	3.5	g/dl
BUN	18.0	mg/dl
Cre	0.77	mg/dl
eGFR	57.8	ml/min/1.73 m^2^
Na	136.5	mEq/L
K	4.6	mEq/L
Cl	101.2	mEq/L
Ca	9.3	mg/dl
Glu	352	mg/dl
HbA1c	7.6	%
CRP	4.07	mg/dl
BNP	2687.1	pg/ml
TSH	1.39	µIU/ml
FT3	1.7	pg/ml
FT4	1.28	ng/dl
ferritin	1902.3	ng/ml
Serum amyroid A	168	µg/ml
sIL-2R	633	IU/ml

ALB, albumin; ALP, alkaline phosphatase; ALT, alanine transaminase; AMY, amylase; APTT, activated partial thromboplastin time; AST, aspartate transaminase; BASO, basophil; BNP, brain natriuretic peptide; BUN, blood urea nitrogen; Ca, calcium; Cl, chloride; CPK, creatine phosphokinase; Cre, creatinine; CRP, C-reactive protein; eGFR, estimated glomerular filtration rate; EO, eosinophil; FT3, free triiodothyronine; FT4, free thyroxine; γ-GTP, γ-glutamyl transpeptidase; Hb, haemoglobin; HbA1c, haemoglobin A1c; INR, international normalized ratio; K, potassium; LDH, lactate dehydrogenase; LYM, lymphocyte; MONO, monocyte; Na, sodium; NEUT, neutrophil; PLT, platelet; PT, prothrombin time; RBC, red blood cell count; sIL-2R, soluble interleukin-2 receptor; T-Bil, total bilirubin; TSH, thyroid-stimulating hormone; TP, total protein; WBC, white blood cell count.

Cardiac amyloidosis (CA) was initially suspected from the ECG and TTE findings. On Day 4, she deteriorated with respiratory distress, cold extremities and non-sustained ventricular tachycardia (NSVT), worsening rapidly despite a dobutamine infusion. Evaluation for CA, including serum free light chains, was negative, whereas cardiac magnetic resonance imaging (MRI) and scintigraphy were precluded by her haemodynamic instability. Emergent right heart catheterization (RHC) revealed postcapillary pulmonary hypertension with a low cardiac index (CI) (*Table 2*), and coronary angiography showed no obstructive coronary artery disease (*Figure 2A–2B*). As the worsening was disproportionate to the suspected CA, endomyocardial biopsy was performed, and rapid histology revealed inflammatory cell infiltration. Anticipating further circulatory failure, we started MCS with veno-arterial extracorporeal membrane oxygenation (ECMO) combined with IMPELLA CP® (ECPELLA) under mechanical ventilation. On Day 8, pulse steroids (methylprednisolone 1000 mg/day for 3 days) were started because CRP and hs-cTnI remained high and TTE showed worsening oedema-like changes in both ventricles (*Figure 2C*). On Day 9, histopathological examination of the myocardium revealed irregular multinucleated giant cells among the inflammatory infiltrate, establishing the diagnosis of GCM (*Figure 3A* and *B*). Prednisolone 60 mg/day was continued thereafter. As she remained device-dependent after one week, the support was escalated to the Impella 5.0®. Pulmonary congestion was improved and she was weaned off MCS on Day 17. Surgical haemostasis was performed at MCS removal. Subsequently, a wound infection developed, and negative pressure wound therapy was initiated. Her steroid dose was reduced to 50 mg/day, but on Day 23, she developed worsening pulmonary congestion with unexplained acute liver dysfunction, prompting a second steroid pulse, as autoimmune activity was considered a possible contributor. Repeat echocardiography showed a LVEF of approximately 35%, indicating incomplete cardiac recovery. Although haemodynamics had stabilized enough to consider device removal, a febrile episode subsequently worsened heart failure. Troponin remained elevated despite a decreasing trend, suggesting corticosteroid therapy may have been insufficient; together, these factors likely contributed to the transient decompensation. After pulse steroid therapy, immunosuppression was resumed with prednisolone 60 mg/day, improving both congestion and liver dysfunction, which were considered related. She was extubated on Day 31, required temporary milrinone for recurrent congestion, and left the intensive care unit (ICU) on Day 44.

**Figure 2:**
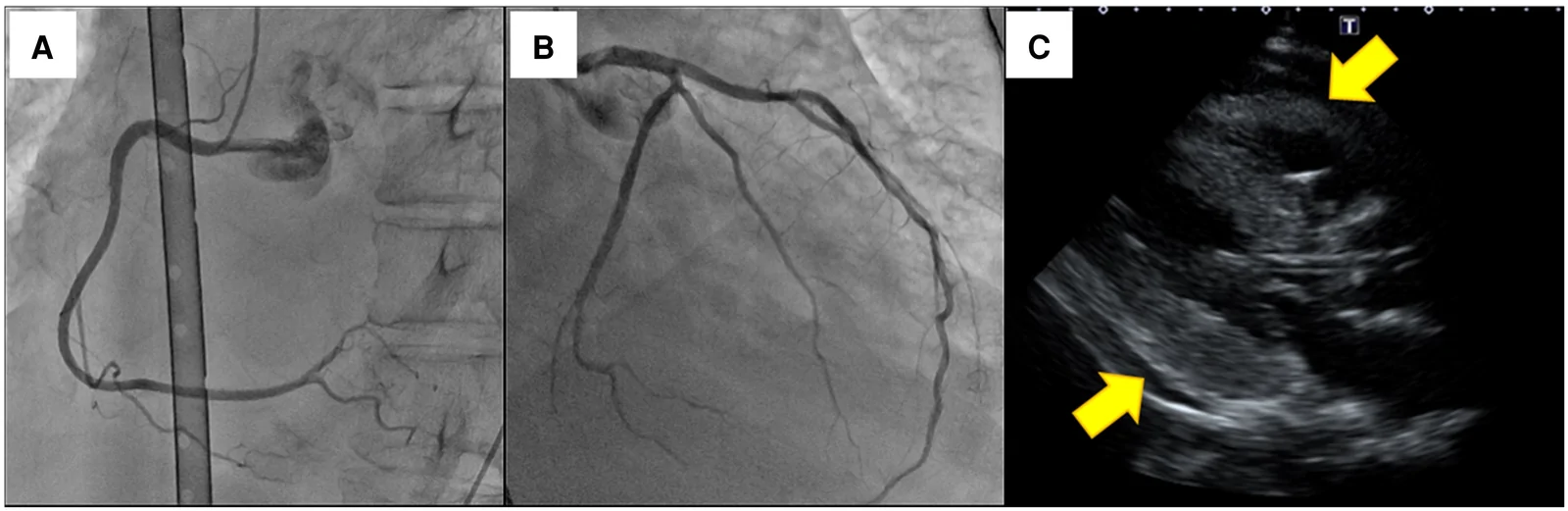
(*A*, *B*). Coronary angiography revealed no significant obstructive coronary artery disease. (*C*). Echocardiography showed a decrease in LVEF to 20% and worsening oedema-like changes in both ventricles.

**Figure 3:**
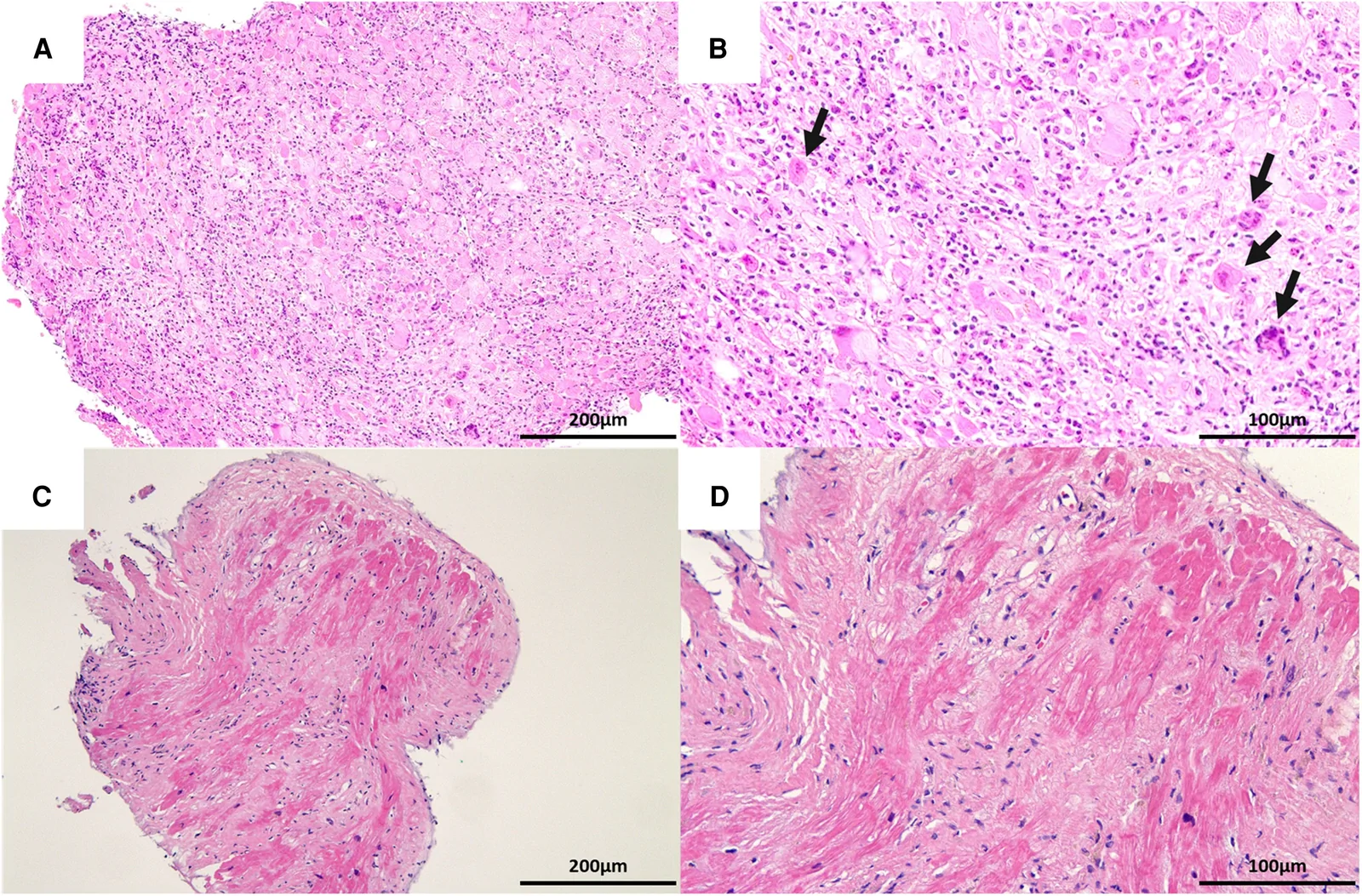
(*A*, *B*). Histopathological findings of the myocardium (haematoxylin and eosin staining). Patchy interstitial inflammatory cell infiltration with associated oedema is observed. Scattered multinucleated giant cells (arrowheads) are present within the inflammatory foci. Cardiomyocyte architecture is largely preserved, with only mild myocyte injury. (*C*, *D*). Pathological examination of the myocardium on Day 151. Extensive myocardial fibrosis was observed, mainly in the perivascular and interstitial regions, with additional involvement of the subendocardium. A mild-to-moderate lymphocytic infiltrate was present; however, the giant cell reaction had resolved. These findings are consistent with the chronic phase of giant cell myocarditis, suggesting significant tissue remodelling and repair.

**Table 2:** RHC pressure study on Days 4 and 151

RHC pressure study	Day 4	Day 66	Day 151
Mean PAWP (mmHg)	31	3	4
PAP (systolic/diastolic [mean], mmHg)	47/30 [38]	17/2 [8]	18/4 [10]
RVP (systolic/end-diastolic, mmHg)	47/15	18/3	21/3
Mean RAP (mmHg)	15	0	1
SvO2/SaO2 (%)	43.2/98.0	−	70.1/99.0
CI (thermodilution method, L/min/m^2^)	1.68	1.88	2.10
CI (Fick method, L/min/m^2^)	1.2	−	1.97

CI, cardiac index; PAP, pulmonary artery pressure; PAWP, pulmonary artery wedge pressure; RAP, right arterial pressure; RHC, right heart catheterization; RVP, right ventricular pressure.

After ICU discharge, inotropes were tapered and discontinued. Steroids were reduced to 50 mg/day on Day 54. On Day 66, RHC showed improved pulmonary hypertension and CI (*Table 2*), repeat histology showed disappearance of giant cells (*Figure 3C* and *D*), and the apical sparing pattern had disappeared on echocardiography. Since the wound infection had healed and she remained stable off inotropes, cyclosporine 100 mg/day was added on Day 69 and the steroid dose reduced to 40 mg/day, then by 5 mg every 2 weeks to 15 mg/day, with cyclosporine levels adjusted to 100–200 ng/ml. Apart from the NSVT episode on Day 4, no sustained ventricular arrhythmia or atrioventricular block was documented. She was discharged on Day 183 after rehabilitation, on prednisolone 15 mg/day, cyclosporine 120 mg/day and guideline-directed medical therapy. Steroids were tapered further during outpatient follow-up, with no relapse in approximately 3 years.

## Discussion

As GCM lacks specific clinical findings, early myocardial biopsy^2^ and early initiation of MCS and immunosuppressive therapy^3^ are key to improving prognosis. Interestingly, in this case the ECG showed low voltage despite LVH on TTE,^5^ and the apical sparing pattern led us to consider CA. However, the rapid progression necessitating MCS precluded MRI and myocardial scintigraphy, and itself indicated the need for myocardial biopsy.^6^ While apical sparing is generally recognized for its high specificity in CA, recent reports suggest it is not strictly pathognomonic: Cotella *et al*.^7^ reported a sensitivity of 72% and specificity of 66%, with apical sparing in 32% of 200 control patients and 6% of healthy subjects. It has also been reported in end-stage renal disease, attributed to myocardial remodelling and haemodynamic burden,^8^ and in hypertensive cardiomyopathy, where acute afterload elevation induced a reversible pseudo-amyloid strain pattern^9^; foreshortening of the left ventricle on inappropriate apical images represents a technical cause.^10^ In this case, the pattern likely resulted from inflammatory infiltration and subsequent oedema being more pronounced in the basal and mid-ventricular regions than in the relatively spared apex.

Although troponin levels were significantly high, CPK-MB was only slightly elevated, and the pathological findings showed largely preserved cardiomyocytes with inflammatory cell infiltration and oedematous changes predominantly in the interstitium. Approximately 5–8% of cellular troponin is unbound in the myocyte cytoplasm,^11^ and irreversible myocyte damage is not a prerequisite for the release of these cytosolic fragments.^12^ The inflammation-associated interstitial oedema may therefore have contributed to myocardial stunning and increased membrane permeability, releasing cytoplasmic troponin despite the absence of extensive structural myocyte disruption.

Two- or three-drug immunosuppression including steroids is recommended for GCM.^1^ However, intensifying immunosuppression under MCS risks infection: our patient's groin wound infection persisted after ECMO removal, and we continued steroids alone until it resolved. Combination therapy therefore warrants caution in patients with GCM on MCS, and gradual escalation guided by cardiac recovery and infection status may be acceptable.

## Lead author biography


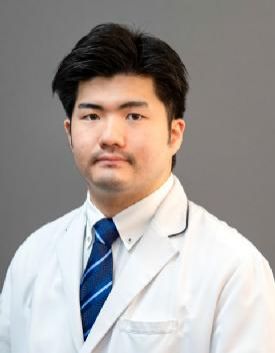


Shun Sawada graduated from Tohoku University in 2019. He completed his residency at Shonan Kamakura General Hospital (2019-2021), followed by a fellowship in Internal Medicine at Iwate Prefectural Central Hospital (2021-2024). He has been a physician in the Department of Cardiology at Shonan Kamakura General Hospital since April 2024. Professional Memberships: Japanese Circulation Society; Japanese Association of Cardiovascular Intervention and Therapeutics; Japanese Heart Failure Society.

## Data Availability

The datasets used and/or analysed during the current study are available from the corresponding author on reasonable request.
